# Genetics of hypertension in populations affected by the Aral Sea ecological crisis: A case-control study

**DOI:** 10.1371/journal.pone.0352877

**Published:** 2026-07-06

**Authors:** Khurshid Ataniyazov, Darya Vladimirovna Zakirova, Khurshid Meylikov, Gulnoz Khamidullaeva, Guzal Abdullaeva, Shahlo Turdikulova, Khurshid Fozilov, Alisher Abdullaev

**Affiliations:** 1 Karakalpakstan regional branch of the Republican specialized scientific and practical medical center of Cardiology, Nukus, Uzbekistan; 2 Center for Advanced Technologies, Tashkent, Uzbekistan; 3 Republican Specialized Cardiological Scientific and Practical Medical Center, Tashkent, Uzbekistan; CUNY School of Medicine: The City College of New York CUNY School of Medicine, UNITED STATES OF AMERICA

## Abstract

**Objective:**

This research investigates the correlation between nine polymorphic variants and the incidence of hypertension and its clinical manifestations in the Ellikqala District in the Republic of Karakalpakstan, Uzbekistan. This district is known as one of the most challenging areas for human habitation due to its high salinity levels in the environment, which contribute to a higher prevalence of cardiovascular diseases.

**Methods:**

This was a population-based case-control study. The study included 801 participants from the Karakalpakstan region of Uzbekistan, of which 621 were patients with essential hypertension, and the remaining 180 were the control group. Genetic analysis was conducted using multiplex qPCR with allele-specific probes. A statistical approach based on logistic regression was applied to identify their association with essential hypertension and its clinical manifestations. Logistic regression under a log-additive genetic model was used to calculate unadjusted (crude) and adjusted odds ratios and 95% confidence intervals. Stratified analyses by sex, age (<60 vs. ≥ 60 years), and BMI (<25 vs. ≥ 25 kg/m²) were performed with Bonferroni correction (k = 18 per stratification; threshold p < 2.8e-3).

**Results:**

The analysis revealed statistically significant associations between essential hypertension and six polymorphic variants after Bonferroni correction (k = 9, corrected threshold: p < 5.6e-3): AGT C521T (rs4762; OR = 0.12, 95% CI: 0.08–0.16, p-Bonferroni = 2.99e-50), AGT T704C (rs699; OR = 0.68, 95% CI: 0.54–0.86, p-Bonferroni = 1.1979e-2), AGTR2 G1675A (rs1403543; OR = 0.52, 95% CI: 0.42–0.64, p-Bonferroni = 4.76e-9), CYP11B2 C(−344)T (rs1799998; OR = 0.47, 95% CI: 0.36–0.60, p-Bonferroni = 8.97e-9), GNB3 C825T (rs5443; OR = 1.66, 95% CI: 1.27–2.18, p-Bonferroni = 1.368e-3), and NOS3 T(−786)C (rs2070744; OR = 0.27, 95% CI: 0.21–0.34, p-Bonferroni = 2.50e-27). NOS3 G894T (rs1799983) showed a nominally significant association prior to correction (OR = 0.68, 95% CI: 0.52–0.89; p = 5.069e-3), but did not survive Bonferroni adjustment (p-Bonferroni = 4.5621e-2) and should be interpreted with caution.

**Conclusion:**

Six out of nine studied polymorphic variants were associated with essential hypertension and its clinical manifestations in the Ellikqala District in the Republic of Karakalpakstan, Uzbekistan.

## Introduction

Globally, cardiovascular diseases (CVD) remain the leading cause of mortality, a trend particularly noticeable in Uzbekistan, where CVD accounted for 61% of deaths in 2023. Essential hypertension, influenced by both genetic predisposition and environmental factors, is a major contributor to this health burden. A lack of detailed genetic data for the region hinders effective diagnosis and the development of tailored preventive and therapeutic strategies [1–3]. The Republic of Karakalpakstan, despite its distinct geographical and ethno-cultural identity, remains largely unexplored in terms of the epidemiology and genetic traits of its population. Within this region, Ellikqala District stands out, located in the northwestern part of Karakalpakstan, Uzbekistan, characterized by its arid landscapes and proximity to the desiccated Aral Sea. The population of the district consists mainly of Uzbeks, Karakalpaks, Turkmens and Kazakhs, representing the wide ethnic diversity of this region [4,5]. The lack of data on the genetic structure of the population and the prevalence of various diseases in the region limits the possibilities for effective diagnosis and the development of adapted preventive and therapeutic approaches [6].

The Aral Sea crisis – one of the most severe human-made ecological disasters of the 20th century – has created a unique setting for studying gene–environment interactions in cardiovascular health [7]. In such conditions of chronic physiological stress, the renin-angiotensin-aldosterone system (RAAS) becomes critically important for maintaining blood pressure [8].

Key genetic components, such as AGT variants (rs699 and rs4762) have established links with hypertension in various populations, including our observations conducted in the capital Tashkent [9]. Variants in angiotensin II receptors (AGTR1 rs5186 and AGTR2 rs1403543) affect receptor sensitivity and have been associated with organ damage [10–12]. Additionally, polymorphisms in GNB3 (rs5443), CYP11B2 (rs1799998), and NOS3 (rs2070744, rs1799983) influence hypertension risk through impacts on aldosterone synthesis, intracellular signaling, and endothelial function [13–15].

However, the combined contribution of these variants to hypertension risk and their interaction with environmental factors – such as saline air, dust storms, and limited access to clean water – remain poorly studied in Uzbekistan. Given the substantial geographic variation in the frequency of relevant polymorphisms, findings from urban cohorts, including our previous study in Tashkent [9,16–19], may not be directly applicable to this environmentally affected region shaped by the Aral Sea disaster. The Republic of Karakalpakstan represents a unique epidemiological setting characterised by chronic environmental exposure to heavy metals, pesticides, and dust from the dried Aral Sea bed; such exposures may interact with genetic susceptibility factors in ways not captured by studies conducted in urban or less environmentally stressed cohorts. This knowledge gap limits the development of population-specific prevention strategies and genetic risk stratification tools for hypertension management in this region. The present study addresses this gap by providing the first comprehensive candidate gene association analysis of hypertension in the Ellikqala district population. We hypothesized that specific SNPs in genes related to the RAAS and endothelial function (AGT, AGTR1, AGTR2, CYP11B2, ADD1, GNB3, NOS3) contribute to essential hypertension susceptibility in this ecologically affected population in ways not fully reflected by existing data. The objective of this study was to evaluate associations between nine candidate SNPs and essential hypertension in a case-control design, and to assess whether these associations remain robust after adjustment for age, sex, and body mass index.

## Methods and materials

This case-control study included 801 participants: 621 patients with essential hypertension, diagnosed according to the ESH/ESC (2018) classification, and 180 normotensive controls. The observed case-to-control ratio of 3.45:1 (621 cases vs. 180 controls) reflects the high prevalence of hypertension and the limited availability of eligible normotensive individuals in the sparsely populated Ellikqala district, where a large proportion of the adult population presented with hypertension or relevant comorbidities. The study was conducted at family clinics in the Ellikqala district of the Republic of Karakalpakstan, Uzbekistan, between February and May 2025. All participants provided written informed consent prior to enrollment. The results of screening of the population over 40 years of age (a total of 2430 respondents) in family clinics of the district were analyzed, according to the modified (for the region of Central Asia and Transcaucasia) WHO PEN protocol. From the total number of respondents, individuals with increased systolic and diastolic blood pressure ≥140 and ≥90 mmHg (SBP and DBP, respectively) were selected, including both newly diagnosed hypertensive patients and those patients who had previously been diagnosed with hypertension but did not control their blood pressure.

### Patient selection

Patient selection criteria included a diagnosis of stage I–III arterial hypertension in individuals of both sexes aged 40–70 years. The study included volunteers of both sexes, and all participants signed written informed consent to participate, which ensured compliance with the ethical standards of the study. Patients with secondary hypertension, severe heart failure (stages III–IV), or advanced coronary artery disease were excluded from the study. Other exclusion factors included cardiac arrhythmias, a history of stroke or myocardial infarction, renal or hepatic insufficiency, and chronic metabolic or oncological diseases. Controls were selected consecutively from normotensive participants (systolic blood pressure <140 mmHg, diastolic blood pressure <90 mmHg, no prior diagnosis of hypertension, and no antihypertensive medication use) identified during the same population screening programme in Ellikqala district. Controls were not formally matched to cases by age or sex; however, inclusion criteria required an age range of 30–72 years, consistent with the case group. The resulting difference in sex distribution between groups (61% female in cases vs. 28% female in controls) is acknowledged as a limitation and addressed through adjusted logistic regression models. Hypertension was diagnosed in accordance with the 2018 ESH/ESC guidelines. All clinical, laboratory, and echocardiographic measurements were obtained using standardized protocols, described in Ataniyazov et al. [6], and were applied identically to cases and controls. All instruments were calibrated and maintained in accordance with the manufacturers’ specifications.

### Genotyping

Total DNA was isolated from venous blood samples with QIAamp DNA Blood Mini Kits (Qiagen, Germany), following the instructions provided. Concentration and purity were verified using Qubit fluorometry (Thermo Fisher Scientific, USA). Polymorphisms were identified by multiplex qPCR with allele-specific primers and DNA probes using the QuantStudio 5 Real-Time PCR System (Applied Biosystems, Foster City, CA, USA). The reaction was carried out using the TaqMan® Genotyping Assay kit (Thermo Fisher Scientific, Waltham, MA, USA) following the manufacturer’s standard protocol. Each reaction contained 10 ng of genomic DNA, 5 μl of TaqMan Genotyping Master Mix, and 0.5 μl of TaqMan Genotyping Assay. Laboratory personnel performing DNA extraction, normalization and subsequent amplification were blinded to the case/control status of all samples. For AGT C521T (rs4762), 24 control samples were excluded from analysis due to insufficient DNA amplification, resulting in a control group size of n = 156 for this locus.

We investigated nine polymorphisms in genes related to blood pressure regulation: *ADD1* rs4961, *AGT* rs699 and rs4762, *AGTR1* rs5186, *AGTR2* rs1403543, *CYP11B2* rs1799998, *GNB3* rs5443, and *NOS3* rs2070744 and rs1799983.

### Statistical analysis

Descriptive statistics for continuous variables were presented as medians and interquartile ranges (IQR: 25th–75th percentiles). The non-normal distribution was confirmed for all continuous variables in both groups; therefore, non-parametric statistical methods were used throughout. Differences between groups were assessed using the Mann-Whitney U test with normal approximation (Z-statistic). To determine the relationship between specific genotypes and the risk of developing hypertension, we employed logistic regression models. Genotype–hypertension associations were analyzed by binary logistic regression under a log-additive genetic model. Primary analyses represent unadjusted (crude) odds ratios. Multivariable adjusted models were additionally fitted for each SNP, incorporating age (<60 vs. ≥ 60, years), sex (men vs. women), and BMI (<24.9 vs. ≥ 25, kg/m^2^) as covariates, based on their known associations with blood pressure and their unequal distribution between case and control groups. Metabolic variables (lipid profile, creatinine, glucose) were not included as covariates in the primary genetic models. To eliminate false-positive results and apply stricter significance criteria for the identified associations, both crude OR and adjusted OR with 95% confidence intervals and Bonferroni-adjusted p-values (k = 9 for 9 SNP comparisons and k = 18 for stratified data comparisons) were calculated and reported in Table 1. All calculations were executed using the SNPassoc package within the R statistical environment (R Core Team, Vienna, Austria).

**Table 1:** Association analysis of polymorphic variants with hypertension in the Uzbek population.

Gene	Polymorphic variant	Risk allele (allele frequency in Uzbek population)	Non risk allele	Log-Additive model							
				Crude model				Adjusted model			
				OR	CI 95%	p-value	p-value, Bonferroni corrected (k=9)	OR	CI 95%	p-value	p-value, Bonferroni corrected (k=9)
*ADD1* G1378T	**rs4961**	T = 0,26	G	0.90	0.69-1.16	0.42022	1.000	0.811	0.607-1.083	0.1551	1.0000
*AGT* C521T	**rs4762**	С = 0.45	Т	0.12	0.08-0.16	**3.321e-51**	**2.99e-50**	0.128	0.090–0.184	**7.64e-29**	**6.88e-28**
*AGT* T704C	**rs699**	С = 0.51	Т	0.68	0.54-0.86	**1.331e-3**	**1.1979e-2**	0.776	0.603–0.999	**4.92e-2**	0.4424
*AGTR1* A1166C	**rs5186**	С = 0.13	А	0.88	0.61-1.25	0.4718	1.000	0.827	0.558–1.223	0.3410	1.0000
*AGTR2* G1675A	**rs1403543**	A = 0.35	G	0.52	0.42-0.64	**5.287e-10**	**4.76e-9**	0.549	0.440–0.685	**1.08e-7**	**9.68e-7**
*CYP11B2* C-344T	**rs1799998**	T = 0.37	C	0.47	0.36-0.60	**9.970e-10**	**8.97e-9**	0.517	0.395–0.676	**1.53e-6**	**1.38e-5**
*GNB3* C825T	**rs5443**	T = 0.26	C	1.66	1.27-2.18	**1.520e-4**	**1.368e-3**	1.668	1.245–2.234	**5.99e-4**	**5.4e-3**
*NOS3* T-786 C	**rs2070744**	T = 0.41	C	0.27	0.21-0.34	**2.779e-28**	**2.50e-27**	0.287	0.219–0.376	**1.36e-19**	**1.22e-18**
*NOS3* G894T	**rs1799983**	Т = 0.20	G	0.68	0.52-0.89	**5.069e-3**	4.5621e-2	0.719	0.537–0.962	**2.61e-2**	0.2353

CI: confidence interval; OR: odds ratio; OR_adj: multivariable logistic regression including age, sex, and BMI as covariates.

### Ethics statement

Ethics approval was received from the Ethics Committee of the Republican Specialized Scientific-Practical Medical Center of Cardiology of Ministry of Health of Uzbekistan (27.01.2025, protocol #2) and was conducted in full accordance with the principles of the Declaration of Helsinki. All study participants provided written informed consent.

## Results

Among hypertensive patients aged 30–70, 382 were female (61.51%) and 239 male (38.49%). In the control group, aged 34–72, there were 50 females (27.78%) and 130 males (72.22%). The Mann–Whitney U test found no significant age difference between hypertensive and control groups (p = 0.09), but BMI differed significantly (p = 1.819e-2, Z = 2.36) (Table 2). Significant differences were also observed in such metabolic parameters as blood glucose, which were higher in the hypertensive group (p = 7.5e-7, Z = 4.95), and triglyceride levels pronouncing difference between cases and controls (p < 1e-16, Z = 13.76) (Table 2). In the Uzbek population, the difference in TC between the group of HTN patients and the control group was statistically significant (p < 1e-16, Z = 11.63) (Table 2). LDL levels were significantly higher in the case group compared to the control (p < 1e-16, Z = 11.32). HDL levels were also significantly different between the two groups (p = 1.214e-4, Z = 3.84) (Table 2). Left Ventricular Mass Index (LVMI) was greater (statistically significant) in the group of hypertensive patients (p = 2.037e-11, Z = 6.7) (Table 2).

**Table 2:** Investigation of the clinical data and medical history according to the Mann-Whitney U tests.

Clinical data	Cases (HTN patients) Median (QL, QU)	Controls (healthy) Median (QL, QU)	Mann-Whitney U	
			Value *P*	Value *Z*
BMI (kg/m^2^)	30 (27, 33)	28 (26, 29)	1.819e-2	2.36
Blood Glucose (mmol/L)	5.2 (5, 6)	4.8 (4, 5)	7.5e-7	4.95
TG (mg/dL)	235 (206, 268)	185 (174, 200)	<1e-16	13.76
TC (mg/dL)	208 (180, 229)	170 (160, 182)	<1e-16	11.63
HDL-C (mg/dL)	41 (38, 45)	40 (36, 42)	1.214e-4	3.84
LDL-C (mg/dL)	121 (104, 132)	93 (85, 103)	<1e-16	11.32
LVMI (g/m^2^)	125 (105, 161.25)	70.5 (63.5, 78)	2.037e-11	6.7

BMI: body mass index; HDL-C: high-density lipoprotein-cholesterol; HTN: hypertension; LDL-C: low-density lipoprotein-cholesterol; LVMI: left ventricular mass index; QL: lower quartile (25%); QU: upper quartile (75%); TC: total cholesterol; TG: triglyceride.

Comparing allele frequencies with global datasets revealed both similarities and differences. For most variants, such as ADD1 rs4961, AGTR1 rs5186, and CYP11B2 rs1799998, minor allele frequencies were comparable to global reports (0.19 vs. 0.26). In contrast, several polymorphisms demonstrated marked deviations. For example, the minor allele frequency of AGT rs4762 (C521T) in our control group was substantially higher (0.54 vs. 0.12), while the frequency of NOS3 rs2070744 (T-786C) was also increased (0.59 vs. 0.28) (Tables 3 and 4).

**Table 3:** Characteristics of studied polymorphic variants and their association with Hypertension in the Uzbek population.

Gene	Polymorphic variant	Location	Molecular consequence	MAF (TOPMed)
*ADD1*	rs4961 (G1378T)	4:2904980	Missense (Gly460Trp)	T = 0.19
*AGT*	rs4762 (C521T)	1:230710231	Missense (Thr174Met)	T = 0.12
*AGT*	rs699 (T704C)	1:230710048	Missense (Met235Thr)	C = 0.46
*AGTR1*	rs5186 (A1166C)	3:148742201	3′UTR variant	C = 0.27
*AGTR2*	rs1403543 (G1675A)	X:116170939	Intronic	A = 0.49
*CYP11B2*	rs1799998 (C-344T)	8:142918184	Promoter/5′ flanking (−344)	С = 0.37
*GNB3*	rs5443 (C825T)	12:6845711	Synonymous (Ser)	T = 0.32
*NOS3*	rs2070744 (T-786C)	7:150992991	Promoter/5′-UTR	C = 0.28
*NOS3*	rs1799983 (G894T)	7:150999023	Missense (Glu298Asp)	T = 0.31

MAF values were obtained from the TOPMed reference panel via dbSNP (NCBI).

**Table 4:** Genotype and allele frequencies in two groups.

Gene	Polymorphic variant	n		Genotype frequency						Allele frequency			
				Common homozygotes		Heterozygotes		Rare homozygotes		Dominant alleles		Recessive alleles	
		case	control	case	control	case	control	case	control	case	control	case	control
*ADD1* G1378T	rs4961	621	180	60.5	53.9	31.6	40.6	7.9	5.6	0.764	0.742	0.237	0.258
*AGT* C521T	rs4762	621	156	82.1	28.2	17.1	34.6	0.8	37.2	0.907	0.455	0.093	0.545
*AGT* T704C	rs699	621	180	37.4	28.9	47.3	45.0	15.3	26.1	0.610	0.514	0.390	0.486
*AGTR1* A1166C	rs5186	621	180	77.8	74.4	21.1	25.0	1.1	0.6	0.883	0.869	0.117	0.131
*AGTR2* G1675A	rs1403543	621	180	40.7	15.6	30.8	38.3	28.5	46.1	0.561	0.347	0.439	0.653
*CYP11B2* C-344T	rs1799998	621	180	28.3	13.9	52.5	45.6	19.2	40.6	0.546	0.367	0.454	0.633
*GNB3* C825T	rs5443	621	180	39.3	51.1	48.0	45.0	12.7	3.9	0.633	0.736	0.367	0.264
*NOS3* T-786 C	rs2070744	621	180	57.3	23.3	35.7	35.6	6.9	41.1	0.752	0.411	0.248	0.589
*NOS3* G894T	rs1799983	621	180	64.3	52.8	30.9	39.4	4.8	7.8	0.797	0.725	0.203	0.275

Results of the logistic regression analysis of polymorphic gene variants in the groups of cases and controls in the Uzbek population demonstrated non-significant association between the ADD1 G1378T (rs4961) variant and HTN (OR = 0.90, 95% CI: 0.69–1.16, p = 0.42022), and AGTR1 A1166C (rs5186) variant and HTN (OR = 0.88, 95% CI: 0.61–1.25, p = 0.4718) (Table 1). Six polymorphic gene variants remained significantly associated with hypertension after Bonferroni correction as well: AGT C521T (rs4762) (OR = 0.12, 95% CI: 0.08–0.16, p-Bonferroni = 2.99e-50), AGT T704C (rs699) (OR = 0.68, 95% CI: 0.54–0.86, p-Bonferroni = 1.1979e-2), AGTR2 G1675A (rs1403543) (OR = 0.52, 95% CI: 0.42–0.64, p-Bonferroni = 4.76e-9), CYP11B2 C-344T (rs1799998) (OR =0.47, 95% CI:0.36–0.60, p-Bonferroni = 8.97e-9), GNB3 C825T (rs5443) (OR = 1.66, 95% CI: 1.27–2.18, p-Bonferroni = 1.368e-3), NOS3 T786C (rs2070744) (OR = 0.27, 95% CI: 0.21–0.34, p-Bonferroni = 2.50e-27). The NOS3 G894T (rs1799983) polymorphism showed a borderline significance, with its corrected p-value being very close to the threshold (OR = 0.68, 95% CI: 0.52–0.89, p = 5.069e-3, p-Bonferroni = 4.5621e-2). Genotype frequency distribution in most groups corresponded to the Hardy-Weinberg equilibrium (p > 0.05), except for the polymorphisms AGT C521T and NOS3 T-786C in the control group and ADD1 G1378T in the case group. For the X-linked AGTR2 G1675A polymorphism, the Hardy-Weinberg equilibrium was assessed only in females. To assess the potential influence of confounding variables, multivariable adjusted logistic regression models were fitted for all nine SNPs under the log-additive model, with age, sex, and BMI included as covariates (Table 1). Five SNPs retained statistical significance after Bonferroni correction in adjusted models: AGT C521T (adj OR = 0.128, 95% CI: 0.090–0.184, p = 6.88e-28), AGTR2 G1675A (adj OR = 0.549, 95% CI: 0.440–0.685, p = 9.68e-7), CYP11B2 C-344T (adj OR = 0.517, 95% CI: 0.395–0.676, p = 1.38e-5), GNB3 C825T (adj OR = 1.668, 95% CI: 1.245–2.234, p = 5.4e-3) and NOS3 T-786C (adj OR = 0.287, 95% CI: 0.219–0.376, p = 1.22e-18). For seven of nine SNPs the adjusted OR differed from the crude OR by less than 15%, confirming robustness of the primary associations.

Stratification by BMI showed that in the group of individuals with a higher BMI (≥25) a set of polymorphisms demonstrated a significant association, and fewer associations were observed in individuals with a BMI below 25. In the BMI ≥ 25 kg/m^2^ subgroup, five polymorphisms remained statistically significant after Bonferroni correction: rs4762 (OR = 0.12, 95% CI: 0.08–0.17, p-Bonferroni = 2.05e-43), rs1403543 (OR = 0.55, 95% CI: 0.44–0.69, p-Bonferroni = 2.86e-6), rs1799998 (OR = 0.47, 95% CI: 0.36–0.62, p-Bonferroni = 3.73e-7), rs5443 (OR = 1.68, 95% CI: 1.26–2.26, p-Bonferroni = 6.3e-3) and rs2070744 (OR = 0.28, 95% CI: 0.22–0.37, p-Bonferroni = 3.96e-22). The polymorphism rs699 reached nominal significance in this subgroup, but did not survive Bonferroni correction (OR = 0.69, 95% CI: 0.54–0.89, p = 3.8e-3, p-Bonferroni = 6.84e-2). In the BMI < 25 kg/m^2^ subgroup, three polymorphisms remained statistically significant after Bonferroni correction: rs4762 (OR = 0.10, 95% CI: 0.04–0.26, p-Bonferroni = 4.59e-7), rs1403543 (OR = 0.36, 95% CI: 0.20–0.65, p-Bonferroni = 5.8e-3) and rs2070744 (OR = 0.18, 95% CI: 0.08–0.40, p-Bonferroni = 2.27e-5). Of note, rs1799998 showed an inverted direction of effect between BMI subgroups: the minor allele was associated with increased hypertension risk in BMI < 25 kg/m^2^ (OR = 2.42), but decreased risk in BMI ≥ 25 kg/m^2^ (OR = 0.47). This pattern should be interpreted with caution given the smaller sample size in the BMI < 25 subgroup (Table 5).

**Table 5:** Association of polymorphisms with hypertension stratified by sex, age and BMI.

Gene/ SNP	Gender	OR	95% CI	p-value	Bonferroni-adjusted p-value (k=18)	Age	OR	95% CI	p-value	Bonferroni-adjusted p-value (k=18)	BMI	OR	95% CI	p-value	Bonferroni-adjusted p-value (k = 18)
*ADD1* rs4961	**Men**	0.94	0.65-1.35	0.737	1.000	**<60 y**	1.04	0.74-1.48	0.807	1.000	**<24.9**	0.88	0.43-1.83	0.7402	1.000
	**Women**	0.73	0.48-1.10	0.141	1.000	**≥60 y**	0.74	0.50-1.09	0.136	1.000	**≥ 25**	0.90	0.68-1.19	0.4615	1.000
*AGT* rs4762	**Men**	0.12	0.08-0.19	**2.26e-31**	**4.07e-30**	**<60 y**	0.09	0.06-0.15	**2.96e-34**	**5.33e-33**	**<24.9**	0.10	0.04-0.26	**2.55e-8**	**4.59e-7**
	**Women**	0.12	0.07-0.22	**3.34e-14**	**6.01e-13**	**≥60 y**	0.15	0.09-0.24	**4.50e-19**	**8.10e-18**	**≥ 25**	0.12	0.08-0.17	**1.14e-44**	**2.05e-43**
*AGT* rs699	**Men**	0.64	0.48-0.87	**3.6e-3**	6.48e-2	**<60 y**	0.74	0.54-1.01	5.5e-2	0.990	**<24.9**	0.55	0.28-1.06	6.97e-2	1.000
	**Women**	1.01	0.66-1.56	0.953	1.000	**≥60 y**	0.61	0.43-0.88	**7.4e-3**	0.1332	**≥ 25**	0.69	0.54-0.89	**3.8e-3**	6.84e-2
*AGTR1* rs5186	**Men**	0.71	0.44-1.14	0.150	1.000	**<60 y**	0.97	0.60-1.55	0.892	1.000	**<24.9**	0.66	0.25-1.73	0.4274	1.000
	**Women**	1.24	0.63-2.46	0.523	1.000	**≥60 y**	0.77	0.44-1.34	0.287	1.000	**≥ 25**	0.91	0.62-1.35	0.6541	1.000
*AGTR2* rs1403543	**Men**	1.87	1.46-2.40	**3.15e-7**	**5.67e-6**	**<60 y**	0.49	0.37-0.65	**5.62e-7**	**1.01e-5**	**<24.9**	0.36	0.20-0.65	**3.22e-4**	**5.8e-3**
	**Women**	0.65	0.43-0.98	**3.97e-2**	0.7146	**≥60 y**	0.55	0.40-0.76	**2.23e-4**	**4.01e-3**	**≥ 25**	0.55	0.44-0.69	**1.59e-7**	**2.86e-6**
*CYP11B2* rs1799998	**Men**	2.38	1.71-3.32	**7.77e-8**	**1.40e-6**	**<60 y**	0.44	0.32-0.62	**6.65e-7**	**1.20e-5**	**<24.9**	2.42	1.16-5.06	**1.41e-2**	0.2538
	**Women**	0.72	0.46-1.10	0.130	1.000	**≥60 y**	0.50	0.34-0.73	**2.95e-4**	**5.31e-3**	**≥ 25**	0.47	0.36-0.62	**2.07e-8**	**3.73e-7**
*GNB3* rs5443	**Men**	1.58	1.11-2.24	**1.0e-2**	0.180	**<60 y**	1.72	1.21-2.44	**1.9e-3**	**3.42e-2**	**<24.9**	1.56	0.77-3.13	0.2037	1.000
	**Women**	1.67	1.04-2.69	**2.86e-2**	0.5148	**≥60 y**	1.62	1.06-2.47	**2.30e-2**	0.4140	**≥ 25**	1.68	1.26-2.26	**3.5e-4**	**6.3e-3**
*NOS3* rs2070744	**Men**	0.22	0.15-0.31	**5.35e-23**	**9.63e-22**	**<60 y**	0.25	0.18-0.35	**4.22e-18**	**7.60e-17**	**<24.9**	0.18	0.08-0.40	**1.26e-6**	**2.27e-5**
	**Women**	0.48	0.31-0.73	**7.8e-4**	**1.40e-2**	**≥60 y**	0.29	0.20-0.43	**1.15e-11**	**2.07e-10**	**≥ 25**	0.28	0.22-0.37	**2.20e-23**	**3.96e-22**
*NOS3* rs1799983	**Men**	0.68	0.49-0.95	**2.41e-2**	0.4338	**<60 y**	0.60	0.42-0.84	**3.9e-3**	7.02e-2	**<24.9**	0.50	0.24-1.05	6.95e-2	1.000
	**Women**	0.93	0.55-1.56	0.786	1.000	**≥60 y**	0.80	0.53-1.21	0.295	1.000	**≥ 25**	0.71	0.54-0.95	**2.12e-2**	0.3816

CI: confidence interval; OR: odds ratio.

Stratification by age after Bonferroni correction (k = 18; threshold p < 2.8e-3) showed, that polymorphisms rs4762, rs1403543, rs1799998 and rs2070744 remained significantly associated with hypertension in both age groups-individuals younger than 60 and those aged 60 and above. However, the rs5443 polymorphism was associated with hypertension in individuals aged 60 and above (OR: 1.72, 95% CI: 1.21–2.44, p-Bonferroni = 3.42e-2), but the association did not survive Bonferroni correction in the younger group (OR: 1.62, 95% CI: 1.06–2.47, p = 2.30e-2, p-Bonferroni = 0.4140). The rs699 polymorphism was associated with hypertension in individuals aged 60 and above (OR: 0.61, 95% CI: 0.43–0.88, p = 7.4e-3), but after Bonferroni correction it lost its statistical significance (p-Bonferroni = 0.1332) (Table 5).

Stratification by gender revealed that rs4762 and rs2070744 polymorphisms retained statistically significant associations with essential hypertension after Bonferroni correction (k = 18; threshold p < 2.8e-3) in both men and women. The rs1403543 and rs1799998 polymorphisms demonstrated significant associations after Bonferroni correction in men only (rs1403543: OR: 1.87, 95% CI: 1.46–2.40, p-Bonferroni = 5.67e-06; rs1799998: OR: 2.38, 95% CI: 1.71–3.32, p-Bonferroni = 1.40e-06). In men subgroup three polymorphisms did not reach statistical significance after Bonferroni correction: rs699 (OR: 0.64, 95% CI: 0.48–0.87, p = 3.6e-3, p-Bonferroni = 6.48e-2), rs5443 (OR: 1.58, 95% CI: 1.11–2.24, p = 1.0e-2, p-Bonferroni = 0.180) and rs1799983 (OR: 0.68, 95% CI: 0.49–0.95, p = 2.41e-2, p-Bonferroni = 6.48e-2). In women subgroup two polymorphisms did not reach statistical significance after Bonferroni correction: rs1403543 (OR: 0.65, 95% CI: 0.43–0.98, p = 3.97e-2, p-Bonferroni = 0.7146) and rs5443 (OR: 1.67, 95% CI: 1.04–2.69, p = 2.86e-2, p-Bonferroni = 0.5148) (Table 5).

## Discussion

In this study, we investigated the associations of several genetic polymorphisms with hypertension in Ellikqala district of the Republic of Karakalpakstan, Uzbekistan, using various clinical parameters and performing stratified analysis by gender, age and BMI. The demographic distribution of hypertensive patients and controls was balanced by age, and no statistically significant differences were observed (p = 0.09). Similarly, differences were observed in metabolic parameters – including weight, glucose, TG level, lipoproteins – and in left ventricular mass index (LVMI), which was markedly higher in case group (Table 2). This discrepancy between cases and controls provides a clinically relevant basis for assessing genotype-phenotype relationships in this study.

As described previously, the Ellikqala region is characterized by relative geographic isolation and unique environmental pressures associated with the Aral Sea crisis, which may promote genetic drift or local inbreeding [20]. Similarly, in our study, although the genotype frequency distribution in most groups conformed to the Hardy-Weinberg equilibrium (p > 0.05), the AGT C521T and NOS3 T-786C polymorphisms in the control groups and the ADD1 G1378T polymorphism in the patient groups deviated. Although we cannot completely exclude the influence of population stratification or potential technical artifacts characteristic of large-scale genotyping [21,22], the consistency of these results in this semi-isolated population suggests a genuine reflection of its genetic architecture.

The distinct allele frequencies observed in the Ellikqala cohort, particularly for the AGT and NOS3 loci, may reflect the unique genetic landscape of Karakalpakstan. Such variations often arise from localized evolutionary processes, including the founder effect or genetic drift, which are characteristic of populations with limited external gene flow [23,24]. Allele frequency distributions do not merely reflect biological variation but also serve as a record of population history, shaped by migration, isolation, and cultural boundaries. Even within Central Asia, populations sharing geography may differ substantially at the genetic level, as shown by Heyer et al. (2009), where within-group variation sometimes exceeded between-group differences [25]. Such patterns underscore that allele frequencies are not fixed attributes but dynamic outcomes of demographic history. In this context, the deviations observed in our study may be viewed not only as technical findings but also as reflections of the complex historical and social processes that have shaped the Uzbek gene pool. These findings highlight the importance of considering population diversity when extrapolating genetic risk estimates derived from global datasets such as TOPMed or gnomAD.

Association analysis showed that several polymorphisms were significantly associated with hypertension also after Bonferroni correction, indicating a strong and independent genetic contribution to the disease and the studied population. In contrast, polymorphisms such as *ADD1 G1378T* and *AGTR1* A1166C did not show significant and reliable associations, despite their reliability confirmed in the world literature [26–28]. While global studies have linked rs1799983 (NOS3) to hypertension in meta-analyses [29], and large-scale candidate-gene research in Japan has implicated rs699 (AGT) and rs1799998 (CYP11B2) in blood pressure regulation [30], these associations have not been consistently replicated across all populations. Our study now demonstrates that in the environmentally stressed population of Ellikqala – where chronic exposure to saline dust and limited healthcare may contribute to the observed associations – these same variants show robust and statistically significant links to clinical hypertension.

In contrast to our Tashkent-based cohort, where associations of AGT and NOS3 variants were limited to subclinical vascular markers, the current study in Ellikqala reveals robust associations between these same polymorphisms and clinical hypertension. One possible interpretation is that people who carry potentially pathogenic gene variants but live in more favorable environmental and social conditions, such as the capital of Uzbekistan, Tashkent, are less prone to clinical manifestations of hypertension, which highlights the well-known relationship between genetic background and environmental exposure. Recent studies report that poor air quality and other environmental pollutants are associated with stronger genetic effects on hypertension, whereas cleaner environments show weaker associations [31–33].

Stratification by BMI showed that some genetic variants were linked to hypertension only in people with BMI ≥ 25. Specifically, SNPs in CYP11B2 (rs1799998) and GNB3 (rs5443) were significant only in this group (Table 5). This pattern may be related to high salt intake – common in Ellikqala due to salty water and limited food choices – which has been associated with altered fluid balance and stronger gene–phenotype associations in previous studies [34–36].

When we divided participants by age (under 60 vs. 60 and older), most genetic variants were linked to hypertension in both groups. However, GNB3 rs5443 was significant only in people under 60 (Table 5), which may suggest that association between HTN and this variant may differ across age strata, possibly reflecting age-related differences in blood pressure regulation [37].

Stratification showed that rs4762 and rs2070744 are associated with hypertension in both sexes, at the same time, the rs1403543 and rs1799998 polymorphisms demonstrated statistically significant association only in men (Table 5). Possible explanations for this difference may include variations in hormonal background, differences in the expression of target genes, as well as interactions with sex-specific environmental factors [38,39].

The study was conducted in the Ellikqala district of Karakalpakstan, a region where cultural diversity, the consequences of the Aral Sea ecological disaster, and the lack of scientific data intersect. The drying up of the Aral Sea has become not only an ecological crisis, but also a humanitarian one, with pollution and public health issues. In conditions where both nature and humans are exposed to long-term adverse factors, understanding genetic characteristics is of particular importance. The results of our study highlight the multifaceted nature of arterial hypertension, which is associated with the combined contribution of genetic, metabolic, and environmental factors. The associations found provide insight into key molecular mechanisms and highlight the need for personalized risk assessment. This study is a valuable contribution to the genetic-epidemiological field and opens up prospects for further study of risk factors for arterial hypertension in Central Asia. However, further work with larger samples and functional analysis of polymorphisms is needed to verify the data obtained and develop prognostic genetic models adapted to specific regions of populations.

### Limitations

Several limitations of this study should be acknowledged. First, the potential for selection bias cannot be excluded: controls were selected consecutively from the same population screening programme and were not formally matched to cases by age or sex, resulting in an unequal sex distribution between groups (61% female in cases vs. 28% female in controls); although adjusted OR are reported, future studies should consider sex-matched recruitment. Second, the case-to-control ratio of 3.45:1, driven by recruitment constraints in the sparsely populated Ellikqala district, may affect the precision of OR estimates, though logistic regression is robust to unequal group sizes and the adjusted OR confirmed the primary findings. Third, residual confounding cannot be fully ruled out: multivariable models adjusted for age, sex, and BMI, but data on dietary sodium intake, antihypertensive medication use among cases, smoking, alcohol consumption, occupational exposures, and socioeconomic indicators were not collected, and no individual-level data on environmental exposure (airborne salts, agrochemical residues, heavy metals) were available – any interpretation of gene–environment interaction therefore remains hypothetical. Fourth, deviations from Hardy-Weinberg equilibrium were observed for AGT C521T and NOS3 T-786C in the control group and for ADD1 G1378T in the case group; while these may reflect genuine population genetics of this semi-isolated region, technical artifacts cannot be entirely excluded. Fifth, stratified analyses by sex, age, and BMI were Bonferroni-corrected within each stratification variable (k = 18) but should still be interpreted as exploratory given the reduced subgroup sample sizes and the absence of correction across all stratification variables simultaneously; in particular, the inverted direction of effect observed for CYP11B2 rs1799998 between BMI subgroups requires confirmation in independent cohorts. Sixth, the study population reflects the specific environmental and demographic context of the Ellikqala district, which limits direct generalizability to other populations. Finally, the case-control design supports the identification of statistical associations but does not establish causality; no functional validation of the identified variants was performed, and replication in an independent cohort is needed.

## Conclusion

In this study, associations were found between polymorphisms: *AGT C521T, AGT T704C, AGTR2 G1675A, CYP11B2 C-344T, GNB3 C825T, NOS3 T-786C* and hypertension in the population of Ellikqala district of Karakalpakstan. Stratification showed that the identified associations may vary depending on various metabolic parameters, which emphasizes the importance of personalized approaches in hypertension therapy. Despite their global significance, some variants (*ADD1 G1378T, AGTR1 A1166C*) did not show any association in this population, which demonstrates the peculiarity of the genetic structure of individual regions and the need for local studies to identify genetic risk factors. These findings enhance our understanding of the complex genetic factors contributing to hypertension in Central Asia, especially in regions with challenging living conditions, where local communities work hard to improve medicine and prevention despite considerable challenges.

## Supporting information

S1 File STROBE Statement — Checklist of items that should be included in reports of case-control studies. (DOCX)
