# Supplementary material for: Genetics of hypertension in populations affected by the Aral Sea ecological crisis: A case-control study
Source: PLoS One. 2026 Jul 6;21(7):e0352877. doi: 10.1371/journal.pone.0352877 (PMC13336188; doi:10.1371/journal.pone.0352877)
Supplement: S1 File — (DOCX) [file pone.0352877.s001.docx]

**STROBE Statement — Checklist of items that should be included in reports of *case-control studies***

*Manuscript: "Genetics of Hypertension in Populations Affected by the Aral Sea Ecological Crisis: A Case-Control Study" — Ataniyazov et al.*

| **Section / Topic** | **Item No** | **Recommendation** | **Reported in revised manuscript** |
| --- | --- | --- | --- |
| **Title and abstract** | 1 | *(a)* Indicate the study's design with a commonly used term in the title or the abstract | Title page ("A Case-Control Study"); Abstract — Methods ("This was a population-based case-control study"). |
|  | 1 | *(b)* Provide in the abstract an informative and balanced summary of what was done and what was found | Abstract — Objective, Methods, Results, and Conclusion paragraphs. |
| **Introduction** | | | |
| **Background / rationale** | 2 | Explain the scientific background and rationale for the investigation being reported | Introduction (paragraphs 1–4): cardiovascular disease burden in Uzbekistan; knowledge gap for Karakalpakstan; environmental context of the Aral Sea region; rationale for studying nine RAAS- and endothelial-function-related SNPs. |
| **Objectives** | 3 | State specific objectives, including any prespecified hypotheses | Introduction — final paragraph: explicit hypothesis (SNPs in RAAS- and endothelial-function genes contribute to essential hypertension susceptibility in this ecologically affected population) and objective (evaluate associations between nine candidate SNPs and essential hypertension, with adjustment for age, sex, and BMI). |
| **Methods** | | | |
| **Study design** | 4 | Present key elements of study design early in the paper | Methods and Materials — opening sentence: "This case-control study included 801 participants: 621 patients with essential hypertension … and 180 normotensive controls." |
| **Setting** | 5 | Describe the setting, locations, and relevant dates, including periods of recruitment, exposure, follow-up, and data collection | Methods and Materials — opening paragraph: family clinics in the Ellikqala district of the Republic of Karakalpakstan, Uzbekistan; recruitment and data collection between February and May 2025; screening of 2,430 adults over 40 years of age under the modified WHO PEN protocol. |
| **Participants** | 6 | *(a)* Give the eligibility criteria, and the sources and methods of case ascertainment and control selection. Give the rationale for the choice of cases and controls | Methods — Patient Selection subsection: cases diagnosed with stage I–III arterial hypertension per 2018 ESH/ESC guidelines, aged 40–70 years; exclusion criteria listed (secondary HTN, severe heart failure, advanced CAD, arrhythmias, prior stroke/MI, renal/hepatic insufficiency, chronic metabolic or oncological disease). Controls were selected consecutively from normotensive participants (SBP < 140 mmHg, DBP < 90 mmHg, no prior HTN diagnosis, no antihypertensive medication use) identified during the same population screening programme. Rationale for case-to-control ratio (3.45:1) explained in Methods opening paragraph. |
|  | 6 | *(b)* For matched studies, give matching criteria and the number of controls per case | Not applicable — this is a non-matched case-control study. The absence of formal matching is explicitly stated in the Patient Selection subsection and acknowledged in the Limitations section. |
| **Variables** | 7 | Clearly define all outcomes, exposures, predictors, potential confounders, and effect modifiers. Give diagnostic criteria, if applicable | Outcome (essential hypertension diagnosed per 2018 ESH/ESC guidelines): Methods — Patient Selection. Exposures (nine candidate SNPs: ADD1 rs4961; AGT rs699, rs4762; AGTR1 rs5186; AGTR2 rs1403543; CYP11B2 rs1799998; GNB3 rs5443; NOS3 rs2070744, rs1799983): Methods — Genotyping subsection; Table 3. Confounders (age, sex, BMI): Methods — Statistical Analysis. Effect modifiers (sex, age, BMI strata): Methods — Statistical Analysis; Results — stratification paragraphs. |
| **Data sources / measurement** | 8* | For each variable of interest, give sources of data and details of methods of assessment (measurement). Describe comparability of assessment methods if there is more than one group | Methods — Patient Selection subsection: blood pressure, anthropometric, and biochemical measurements (TC, TG, HDL-C, LDL-C, glucose, BUN, creatinine), echocardiographic LVMI, and microalbuminuria were obtained using standardized protocols described in Ataniyazov et al. (ref. 6); all instruments were calibrated and maintained per manufacturers' specifications; identical protocols were applied to cases and controls. Methods — Genotyping subsection: DNA isolation (QIAamp DNA Blood Mini Kits), purity verification (Qubit), genotyping by multiplex qPCR with TaqMan assays on the QuantStudio 5 Real-Time PCR System. |
| **Bias** | 9 | Describe any efforts to address potential sources of bias | Laboratory personnel performing DNA extraction, normalization and amplification were blinded to case/control status (Methods — Genotyping). Controls were drawn consecutively from the same screening programme as cases to reduce selection bias. Multivariable logistic regression adjusted for age, sex, and BMI was performed for all SNPs to address residual confounding (Methods — Statistical Analysis). Sources of bias not fully addressed by design (sex imbalance, unmeasured environmental exposures, residual confounding) are explicitly acknowledged in the Limitations section. |
| **Study size** | 10 | Explain how the study size was arrived at | Methods — opening paragraph: 2,430 adults over 40 years of age were screened under the modified WHO PEN protocol; 801 individuals met inclusion criteria (621 cases, 180 controls). The 3.45:1 case-to-control ratio reflects the high local prevalence of hypertension and the limited availability of eligible normotensive individuals in the sparsely populated Ellikqala district. The case-to-control ratio and its potential effect on precision are discussed in the Limitations section. |
| **Quantitative variables** | 11 | Explain how quantitative variables were handled in the analyses. If applicable, describe which groupings were chosen and why | Methods — Statistical Analysis subsection: continuous variables summarized as medians and IQRs (25th–75th percentiles); non-normality confirmed for all continuous variables, justifying use of non-parametric tests (Mann–Whitney U). For stratified analyses, age was dichotomized at 60 years and BMI at 25 kg/m² — categorizations consistent with conventional clinical thresholds for cardiovascular risk stratification. |
| **Statistical methods** | 12 | *(a)* Describe all statistical methods, including those used to control for confounding | Methods — Statistical Analysis: Mann–Whitney U test (Z-statistic) for between-group comparisons of continuous variables; binary logistic regression under a log-additive genetic model for SNP–HTN associations, reporting both crude and multivariable adjusted ORs (adjustment for age, sex, BMI) with 95% CIs; Bonferroni correction applied to control the family-wise false-positive rate (k = 9 for the primary analysis, threshold p < 5.6 × 10⁻³); analyses conducted with the SNPassoc package in R. |
|  | 12 | *(b)* Describe any methods used to examine subgroups and interactions | Methods — Statistical Analysis: stratified analyses by sex (men vs. women), age (<60 vs. ≥60 years), and BMI (<25 vs. ≥25 kg/m²), each under the log-additive model with Bonferroni correction within each stratification variable (k = 18; threshold p < 2.8 × 10⁻³). Subgroup results reported in Table 5 and in the Results section. |
|  | 12 | *(c)* Explain how missing data were addressed | Methods — Genotyping subsection: complete-case analysis at each locus. For AGT C521T (rs4762), 24 control samples were excluded from analysis of that locus only owing to insufficient DNA amplification (n = 156 controls for this SNP); no other missing data were identified, and no imputation was performed. |
|  | 12 | *(d)* If applicable, explain how matching of cases and controls was addressed | Not applicable in the design sense — controls were not formally matched. The resulting baseline imbalance (notably sex distribution: 61% female in cases vs. 28% in controls) was addressed analytically via multivariable adjustment for age, sex, and BMI (Methods — Statistical Analysis) and is acknowledged as a limitation. |
|  | 12 | *(e)* Describe any sensitivity analyses | Results — regression paragraph: comparison of crude and covariate-adjusted ORs serves as a sensitivity analysis for confounding by age, sex, and BMI; for seven of nine SNPs the adjusted OR differed from the crude OR by less than 15%, confirming the robustness of the primary associations. |
| **Results** | | | |
| **Participants** | 13* | *(a)* Report numbers of individuals at each stage of study — eg numbers potentially eligible, examined for eligibility, confirmed eligible, included in the study, completing follow-up, and analysed | Methods — opening paragraph and Genotyping subsection; Results — opening paragraph. 2,430 individuals were screened; 801 met inclusion criteria and were enrolled (621 cases, 180 controls); 24 control samples were excluded from analysis of AGT C521T due to insufficient DNA amplification, leaving n = 156 controls for that locus only; all other analyses used the full set of 801 participants. |
|  | 13* | *(b)* Give reasons for non-participation at each stage | Methods — Patient Selection subsection: exclusion criteria specified for both cases (secondary HTN, severe heart failure, advanced CAD, arrhythmias, prior stroke/MI, renal/hepatic insufficiency, chronic metabolic or oncological disease) and controls (prior HTN diagnosis or current antihypertensive use). Genotyping-related exclusions for AGT C521T detailed in the Genotyping subsection. |
|  | 13* | *(c)* Consider use of a flow diagram | A flow diagram was not included. Participant numbers at each stage are reported in the text (Methods — opening paragraph and Genotyping subsection; Results — opening paragraph). |
| **Descriptive data** | 14* | *(a)* Give characteristics of study participants (eg demographic, clinical, social) and information on exposures and potential confounders | Results — opening paragraph and Table 2: age, sex distribution, BMI, fasting blood glucose, lipid profile (TC, TG, HDL-C, LDL-C), and LVMI reported separately for cases and controls. Genotype and allele frequencies for all nine SNPs in cases and controls reported in Table 4. |
|  | 14* | *(b)* Indicate number of participants with missing data for each variable of interest | Methods — Genotyping subsection and Table 4 footnote: AGT C521T (rs4762) — n = 156 controls (24 excluded for insufficient DNA amplification); all other SNPs and all clinical/biochemical variables — complete data for the full sample. |
| **Outcome data** | 15* | Report numbers in each exposure category, or summary measures of exposure | Results — Table 4: genotype frequencies (common homozygote, heterozygote, rare homozygote) and allele frequencies for all nine SNPs in cases and controls. Hardy–Weinberg equilibrium assessment reported in Results — regression paragraph (deviations observed for AGT C521T and NOS3 T-786C in controls and for ADD1 G1378T in cases). |
| **Main results** | 16 | *(a)* Give unadjusted estimates and, if applicable, confounder-adjusted estimates and their precision (eg, 95% confidence interval). Make clear which confounders were adjusted for and why they were included | Results — regression paragraph and Table 1: both crude and adjusted ORs with 95% CIs reported for all nine SNPs under the log-additive model, with raw and Bonferroni-corrected p-values. Confounders (age, sex, BMI) and the rationale for their inclusion stated in Methods — Statistical Analysis subsection. |
|  | 16 | *(b)* Report category boundaries when continuous variables were categorized | Methods — Statistical Analysis subsection and Results — stratification paragraphs: age dichotomized at 60 years (<60 vs. ≥60); BMI dichotomized at 25 kg/m² (<25 vs. ≥25); sex coded as men vs. women. |
|  | 16 | *(c)* If relevant, consider translating estimates of relative risk into absolute risk for a meaningful time period | Not applicable — odds ratios from a case-control design; absolute-risk translation requires longitudinal data not available in the present study. |
| **Other analyses** | 17 | Report other analyses done — eg analyses of subgroups and interactions, and sensitivity analyses | Results — stratification paragraphs (BMI, age, sex) and Table 5: stratified analyses with Bonferroni correction (k = 18; threshold p < 2.8 × 10⁻³). Sensitivity check via crude-versus-adjusted OR comparison reported in Results — regression paragraph. The inverted direction of effect for CYP11B2 rs1799998 between BMI subgroups is flagged in the Results and addressed in the Limitations. |
| **Discussion** | | | |
| **Key results** | 18 | Summarise key results with reference to study objectives | Discussion — opening paragraph and paragraph on association analysis: six SNPs (AGT C521T, AGT T704C, AGTR2 G1675A, CYP11B2 C-344T, GNB3 C825T, NOS3 T-786C) significantly associated with essential hypertension after Bonferroni correction; NOS3 G894T borderline; ADD1 G1378T and AGTR1 A1166C not associated. |
| **Limitations** | 19 | Discuss limitations of the study, taking into account sources of potential bias or imprecision. Discuss both direction and magnitude of any potential bias | Discussion — Limitations subsection: selection bias (consecutive non-matched control sampling; 61% vs. 28% female imbalance); case-to-control ratio of 3.45:1 (effect on precision); residual confounding (unmeasured dietary sodium, antihypertensive medication use, smoking, alcohol, occupational exposures, socioeconomic indicators; no individual-level environmental exposure data); deviations from Hardy–Weinberg equilibrium; exploratory nature of stratified analyses despite within-stratum Bonferroni correction; CYP11B2 rs1799998 effect-direction inversion between BMI subgroups; limited generalizability; case-control design supports association but not causation; lack of functional validation and external replication. |
| **Interpretation** | 20 | Give a cautious overall interpretation of results considering objectives, limitations, multiplicity of analyses, results from similar studies, and other relevant evidence | Discussion — multiple paragraphs: results interpreted against findings from our previous Tashkent cohort, from neighbouring Central Asian populations (Kazakh, Yakut), and from global meta-analyses; deviations interpreted in light of population history (founder effects, drift); causal language replaced with association language throughout; environmental modifiers framed as a hypothesis to be tested rather than as an established mechanism. |
| **Generalisability** | 21 | Discuss the generalisability (external validity) of the study results | Discussion — Limitations subsection: "the study population reflects the specific environmental and demographic context of the Ellikqala district, which limits direct generalizability to other populations." Allele frequencies and effect sizes observed here may not transfer to urban Uzbek cohorts or to populations outside the Aral Sea region. |
| **Other information** | | | |
| **Funding** | 22 | Give the source of funding and the role of the funders for the present study and, if applicable, for the original study on which the present article is based | Funding information has been removed from the body of the manuscript in accordance with PLOS ONE policy and is provided exclusively in the Funding Statement field of the online submission form. |

*Give information separately for cases and controls.

**Note:** An Explanation and Elaboration article discusses each checklist item and gives methodological background and published examples of transparent reporting. The STROBE checklist is best used in conjunction with this article (freely available on the websites of PLOS Medicine, Annals of Internal Medicine, and Epidemiology). Information on the STROBE Initiative is available at http://www.strobe-statement.org.
